# Preventing common mental health problems by increasing neighbourhood socioeconomic status: a mental health impact assessment in Rotterdam, the Netherlands

**DOI:** 10.1093/eurpub/ckae222

**Published:** 2025-01-13

**Authors:** Daina Kosīte, Frank J van Lenthe, Mark J Nieuwenhuijsen, Mariëlle A Beenackers

**Affiliations:** Department of Public Health, Erasmus MC, University Medical Center Rotterdam, Rotterdam, the Netherlands; Department of Public Health, Erasmus MC, University Medical Center Rotterdam, Rotterdam, the Netherlands; ISGlobal, Barcelona, Spain; Department of Public Health, Erasmus MC, University Medical Center Rotterdam, Rotterdam, the Netherlands

## Abstract

Neighbourhood socioeconomic status (NSES) has been identified as a determinant of mental health. In this study, we aimed to quantify how many cases of common mental health problems could be prevented by increasing NSES in the most socioeconomically deprived neighbourhoods in Rotterdam, the Netherlands, and how the increases in NSES would affect mental health inequalities. We used publicly available data for conducting a quantitative Health Impact Assessment (HIA) of two counterfactual policy scenarios. In Scenario 1, we set the NSES to the Rotterdam median score for all neighbourhoods which were below the Rotterdam median. In Scenario 2, we set the NSES score to the Dutch national average socioeconomic status score for the neighbourhoods that were below the national average. We estimated that Scenario 1 could prevent 5847 (95% CI, 2700–7999) or 10.7% of annual cases of common mental health problems, and Scenario 2 could prevent 10 713 (95% CI, 4875–14 799) or 19.6% of annual cases in Rotterdam while also reducing mental health inequalities between neighbourhoods in both scenarios. Given the substantial improvements in population mental health that enhanced neighbourhood socioeconomic conditions would bring, policy implementation is urgently needed.

## Introduction

Mental health problems are among the leading causes of disability worldwide. Amidst all mental illnesses, depression and anxiety are the most common, jointly affecting 581 000 lives globally [1]. Alarmingly, the number of individuals affected by mental health problems has grown by 48% between 1990 and 2019, indicating that the current approaches of prevention and treatment are insufficient and that there is an urgent need for more effective strategies to address mental health problems [2]. At the same time, urbanization is on the rise as more and more people are relocating to live in the cities. It has been estimated that in 2018, approximately 55% of the population worldwide lived in urban areas, and by 2050, the urban population is projected to increase to 68% [3]. Previous research shows that mental health problems are especially common among city residents; therefore, approaches for urban mental health are crucial to prevent further incidence of mental health problems [4].

A substantial body of literature indicates that neighbourhood socioeconomic status (NSES) in urban areas plays a role in mental health [5–7]. A study that pooled the data from eight Dutch cohort studies revealed that NSES is associated with mental health in all of the included cohorts [6]. NSES is determined by several factors, most commonly encompassing average household income, educational attainment of residents, and local employment rates [7]. Furthermore, several studies highlight that mental health inequalities exist between lower and higher socioeconomic status (SES) neighbourhoods—individuals living in lower SES neighbourhoods typically have more mental health problems as compared to the residents of higher SES areas [7]. Several mechanisms have been proposed to explain the association between mental health and NSES. Previous studies suggest that inequality in mental health outcomes between neighbourhoods can partially be explained by social composition, i.e. that disadvantaged individuals more commonly live in low SES neighbourhoods. Indeed, some studies suggest that individual-level SES plays a key role in explaining the association between NSES and mental health [8]. Other research suggests that certain neighbourhood contextual factors contribute to mental health over and above the individual-level characteristics [9]. A key pathway includes social-interactive mechanisms, such as social capital [10], social cohesion [11], collective efficacy to make changes in one’s neighbourhood [12], and trust between people in neighbourhoods [13], suggesting that these social characteristics may mediate the relationship between NSES and mental health [14]. Also, the neighbourhood physical environment has been shown to affect mental health, in particular the quality of housing [15]. Previous research and attempts to theorize the links between NSES and mental health suggest that it is a rather complex and multifaceted relationship that cannot be explained by a single mediating factor [14]. Furthermore, while increasing the living standards of their residents is on the agenda for many cities around the world [16], the existing research leaves a gap in our understanding regarding the potential reduction of mental health issues that would occur through the enhancement of NSES in a specific population, for example, in a specific city or a country.

Rotterdam provides a relevant case study for this investigation because it has the lowest average SES among the four major cities in the Netherlands, and a substantial inequality exists between Rotterdam neighbourhoods in terms of area socioeconomic deprivation [17]. For several decades, Dutch urban policies have focused on the social mixing approach to ensure that residents do not segregate in specific neighbourhoods based on their socioeconomic characteristics [18]. The social mixing approach means that the NSES is changed by stimulating changes in the composition of the residents, e.g. attracting more high-income households or preventing low-income households from residing in certain areas. In the context of mental health, it should be made explicit that displacing low SES residents to achieve a better average NSES score would arguably not result in improved mental health outcomes overall in the city [19]. Based on prior reports on the scale of socioeconomic problems in the south neighbourhoods of Rotterdam, the National Program Rotterdam South was issued in 2011. The programme is primarily designed to support the residents of Rotterdam South rather than to restructure the neighbourhoods [20]. The National Programme Rotterdam South aims to ensure that, within 20 years, Rotterdam South has comparable outcomes in terms of school performance, employment, and housing to the average of the four main cities in the Netherlands. The programme integrates various interventions on education, economic strengthening, and improvements in the physical quality of the neighbourhoods with the ultimate aim of improving the neighbourhood socioeconomic conditions in the area [21].

In the current study, we aim to apply the insights from previous literature regarding the impact of NSES on mental health to the population of Rotterdam to ultimately estimate how many cases of depression and anxiety could be prevented each year by increasing the NSES in the most socioeconomically deprived areas of the city. We therefore use the Health Impact Assessment (HIA) framework [22] in a novel way to model two scenarios of varying levels of NSES increases to estimate the impact on the prevalence of common mental health problems and mental health inequalities between neighbourhoods.

## Methods

### Health impact assessment framework

This study was reviewed by the Medical Ethics Committee at the Erasmus MC and deemed not to be human-subject research since only publicly available data were used. We conducted a quantitative HIA based on the comparative risk assessment framework [22]. The HIA methodology offers a possibility to translate scientific findings about the relationship between exposure and outcome into tangible real-life impact on specific populations. Traditionally, this approach is used for assessing the health impact of changes in environmental exposures. For example, studies showed how many cases of mental health problems could be prevented by increasing green space [23] or how many deaths could be prevented by reducing urban air pollution [24]. In the present study, we aimed to assess the impact of changes in socioeconomic determinants of mental health by setting two counterfactual scenarios for the NSES changes in Rotterdam and calculated the number of depression and anxiety cases that could be prevented in each of the scenarios.

### Population description

The study population was comprised of all Rotterdam residents above the age of 18. In 2016, that included 505 256 individuals. Population data were sourced from the open access database of the Rotterdam municipality [25].

### Spatial unit

The analysis was carried out for Rotterdam neighbourhood administrative units. In 2016, there were 92 neighbourhoods in Rotterdam. However, we excluded industrial areas with very few or no residents (*n* = 14), which resulted in a total of 78 neighbourhoods that were included in the analysis.

### Measures

#### Common mental health problems (depression and anxiety)

Data on the prevalence of common mental health problems are publicly available on the neighbourhood level for all Dutch municipalities, including Rotterdam [26]. Common mental health problems were assessed in a representative sample of adults as a part of the Health Monitor survey that is collected every 4 years by the Dutch health authorities. In 2016, 460 000 individuals were invited to participate in the survey, and approximately 7000 (1.5%) completed the survey [27]. The answers to the Kessler-10 (K-10) Psychological Distress Scale [28] are summarized in a score between 10 and 50, and a score of 30–50 points indicates high risk of an anxiety disorder or depression. The Dutch health authorities used the XGBoost method to provide the model-based percentage of inhabitants at high risk of common mental health problems per neighbourhood [29]. In the current study, we estimated the number of residents experiencing mental health problems by multiplying the percentage of adult residents at high risk of an anxiety disorder or depression (scoring 30–50 points on K-10) with the number of adult residents per neighbourhood.

#### Neighbourhood socioeconomic status score

The NSES score we used in our analysis was developed by the Netherlands Institute of Social Research and has been widely used in research and was accessed via the GECCO database [30]. The NSES score is a composite score based on average income, percentage of residents with low education attainment, low income, and percentage of unemployed residents in the neighbourhood as determined by a principal component analysis. The score is centred around zero, meaning that 0 points reflect the national average NSES score, a negative score indicates lower NSES, and a positive score indicates higher than the national average NSES score. SES scores were made available as averages per four-digit postal-code area, which is a different spatial unit than the neighbourhood administrative unit that we used in our analysis. Therefore, the NSES score required data pre-processing by a Geographical Information Systems (GIS) expert to apply area proportional transformation of polygon attribute data between administrative units of different sizes and shapes.

### Exposure-response function

An exposure-response function (ERF) refers to the relationship between exposure and outcome that has been established in previous research. Ideally, the ERF is based on a pooled estimate or meta-analysis instead of a single previous study. In our case, the ERF was obtained from a study by Generaal *et al.* that combined eight Dutch cohort studies and estimated that with one point increase in the Dutch NSES score, the odds ratio of depression is 0.90 [95% confidence interval (CI) 0.87–0.95] [6]. Since using comparative risk assessment for socioeconomic exposures is a novel application of the method, there is a lack of reliable estimates of relative risk that is usually preferred for the ERF. In this first attempt, we used odds ratio as an approximation for the relative risk. This approach has been utilized in previous studies [23] and rests on the assumption that for rare conditions (i.e. less than 10% prevalence), the value of odds ratio is close to the value of relative risk [31].

### Procedure

Firstly, population data were linked with data on NSES and the number of residents at risk of common mental health problems per neighbourhood. We used descriptive statistics to analyse and visualize the NSES and mental health indicators, their spatial distribution across the neighbourhoods of Rotterdam, and relationship between the exposure and outcome. We used the R package ggplot2 for data visualizations, including for creating maps of Rotterdam [32]. We followed the HIA procedure based on the UTOPHIA tool [22] (see Supplementary Fig. S1), where firstly, exposure difference was calculated between the current and each counterfactual scenario (Step 1). Then, using the function between exposure and outcome from previous literature [6], the relative risk was scaled for the exposure difference within each neighbourhood (Step 2). Population Attributable Fraction (PAF) was calculated to estimate the proportion of mental health problems that can be attributed to the exposure level difference in each neighbourhood (Step 3). Finally, by multiplying the PAF with the number of residents at high risk of depression and anxiety, attributable cases were estimated in each neighbourhood and in Rotterdam overall (Step 4). The procedure was repeated with lower and upper bounds of the 95% CI of the ERF to get an indication of the degree of uncertainty of the point estimate. Additionally, we calculated the absolute and relative mental health inequalities between the neighbourhoods at baseline and in each of the two counterfactual scenarios.

### Counterfactual scenarios

In order to study the impact that increasing the SES of Rotterdam’s most deprived neighbourhoods would have on mental health, we set out two potential scenarios. Our aim was to illustrate the effects of changes in the exposure of various magnitudes.

#### Scenario 1: the most socioeconomically deprived neighbourhoods in Rotterdam reaching the median socioeconomic status of Rotterdam

This counterfactual scenario is closely linked with the aim of Rotterdam municipality to reduce inequalities between neighbourhoods of the city. In this scenario, we model the increase of NSES score to the Rotterdam median score (−0.9 SES score) as a minimum.

#### Scenario 2: all neighbourhoods in Rotterdam currently below the national average reaching the national average socioeconomic status

The second scenario offers a more ambitious estimate by looking at the impact of increasing the NSES score to the national average of 0 points as a minimum. This means that in the neighbourhoods that previously scored lower than 0, the NSES value was set to 0 points. This scenario allows us to envision the currently most socioeconomically deprived areas of Rotterdam as average neighbourhoods for the Dutch standards.

## Results

### Baseline statistics

**Figure 1.**
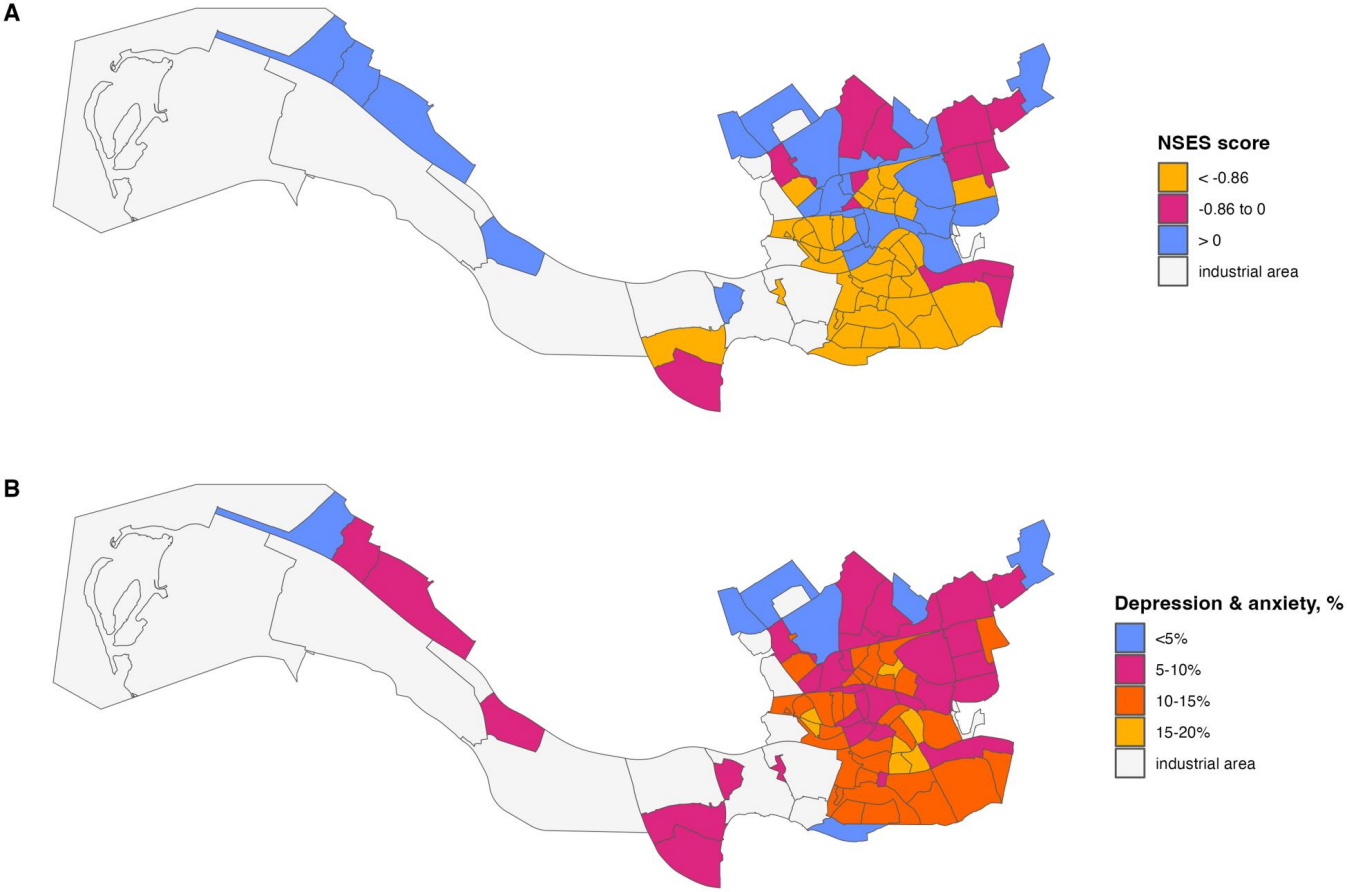
Distribution of the NSES scores (A) and prevalence of residents at high risk of depression and anxiety (B) across the neighbourhoods of Rotterdam.

The Rotterdam median neighbourhood SES score is −0.9 points, and it varies between −4.3 in the most socioeconomically deprived neighbourhood and 1.9 in the neighbourhood with the highest SES. Figure 1A shows the distribution of the NSES scores across the neighbourhoods of Rotterdam. It is estimated that in 2016, approximately 10.8% of Rotterdam adult residents were at high risk of depression and anxiety (*n* = 54 681). The percentage of residents at high risk of mental health problems varies between the neighbourhoods from 3.6% to 19.7% (see Fig. 1B). Consistently with the previous literature, NSES is associated with the prevalence of common mental health problems (see Supplementary Fig. S2).

### Health impact assessment

**Figure 2.**
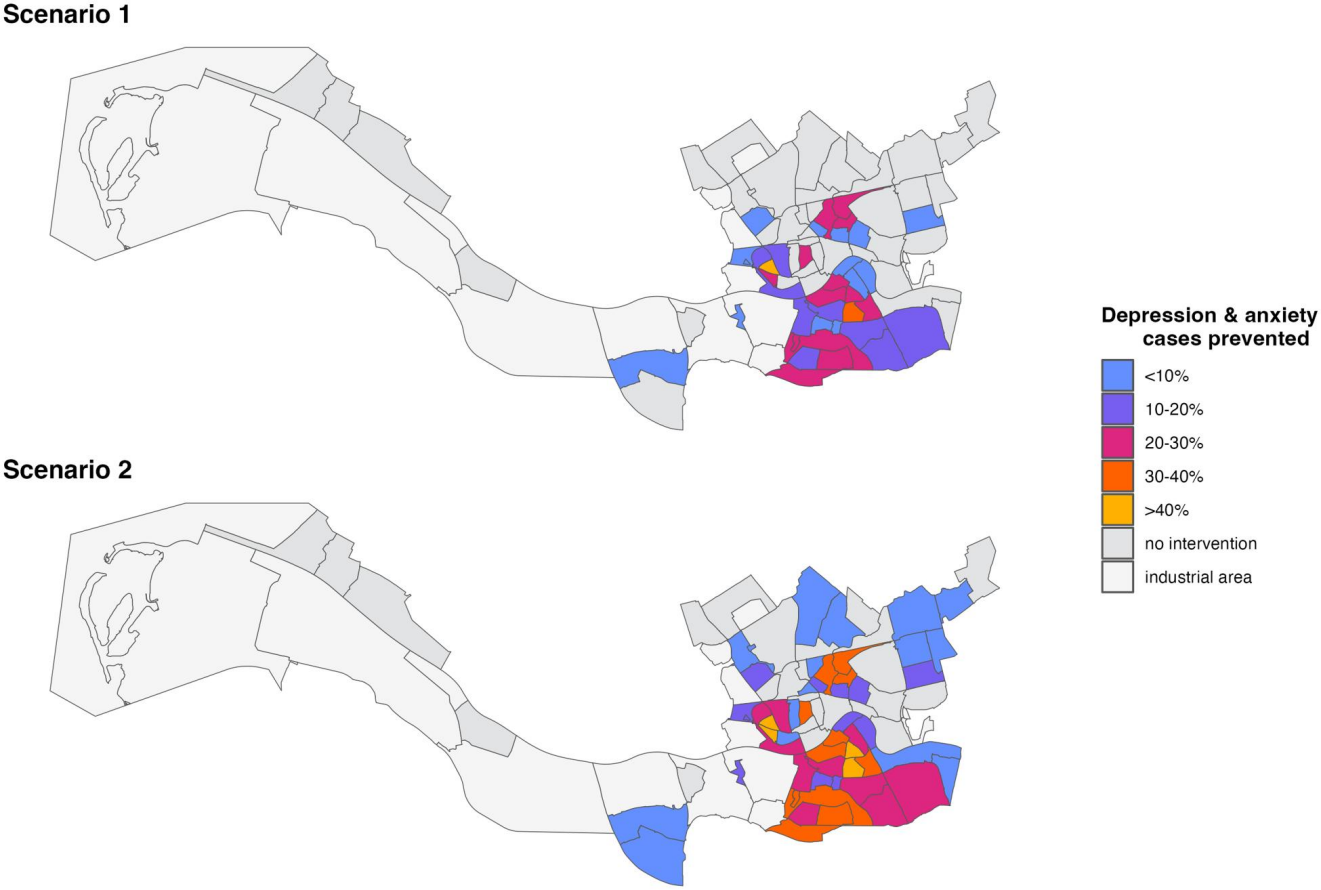
Results of the mental health impact assessment showing the percentage of attributable cases (i.e. depression and anxiety cases that could be prevented by improved NSES score) per neighbourhood.

We estimated that the following number of depression and anxiety cases could be prevented yearly based on the two scenarios: 5847 (95% CI, 2700–7999) cases in Scenario 1 and 10 713 (95% CI, 4875–14 799) cases in Scenario 2 (Table 1). In the first scenario, by increasing the NSES in the 39 currently most socioeconomically deprived neighbourhoods in Rotterdam to the current Rotterdam median, 10.7% of the individuals each year experiencing depression and anxiety could be spared from these common mental health problems. Scenario 2, where Rotterdam neighbourhoods scoring below the national average SES (*n* = 53) would reach the national average, could prevent 19.6% of depression and anxiety cases. Figure 2 shows the depression and anxiety cases that could be prevented per neighbourhood in Rotterdam.

**Table 1.** Attributable cases of depression and anxiety for each of the counterfactual scenarios

	Number of neighbourhoods improved	Mean NSES change	Attributable cases (95% CI)	Attributable cases, % of the total cases
Scenario 1	39	1.3	5847 (2700–7999)	10.7
Scenario 2	53	1.7	10 713 (4875–14 799)	19.6

We analysed the impact of the two counterfactual scenarios on the absolute and relative mental health inequalities between neighbourhoods. In Scenario 1, the absolute inequality in mental health between the 39 neighbourhoods above the Rotterdam NSES median and 39 neighbourhoods below the median would be reduced from 5.2 to 3.1 percentage points. We estimated that at the baseline, the most socioeconomically deprived neighbourhoods (*n* = 39) had 1.7 times higher prevalence of mental health problems than the more affluent neighbourhoods, while following the intervention, this relative inequality would reduce to 1.4. In Scenario 2, the absolute inequality between the neighbourhoods below (*n* = 53) and above (*n* = 25) the national average SES score would be reduced by from 5.5 to 4.0 percentage points. The relative inequality would reduce from 1.9 to 1.6 in Scenario 2 (Table 2).

**Table 2.** Absolute and relative mental health inequalities between neighbourhoods that would experience increases in NSES (intervention neighbourhoods) vs. neighbourhoods that would remain the same (no intervention neighbourhoods) in the two counterfactual scenarios

		Prevalence of mental health problems, %		Absolute inequality	Relative inequality
		No intervention neighbourhoods	Intervention neighbourhoods^a^		
Scenario 1	Pre-intervention	8.0	13.2	5.2	1.7
	Post-intervention	8.0	11.1	3.1	1.4
Scenario 2	Pre-intervention	6.5	12.0	5.5	1.9
	Post-intervention	6.5	10.5	4.0	1.6

a In Scenario 1, intervention is implemented in 39 neighbourhoods; in Scenario 2, intervention in implemented in 53 neighbourhoods.

## Discussion

The results presented in this study highlight the potential positive impact on mental health that could result from enhancing the SES of the most socioeconomically deprived neighbourhoods of Rotterdam. If the most deprived neighbourhoods in Rotterdam reached the Rotterdam median SES score, 10.7% of depression and anxiety could be prevented. If there were no neighbourhoods in Rotterdam that score below the national average SES, then 19.6% of depression and anxiety cases could be avoided. With the two distinct scenarios, we illustrated that as NSES increases, the extent of mental health enhancements for residents also increases. Additionally, both scenarios would lead to a considerable reduction in mental health inequalities between neighbourhoods.

Given the limitations of the HIA methodology, we are unable to distinguish through which mechanisms exactly the enhanced population mental health would be achieved. However, our findings illustrate that population mental health strategies could extend beyond healthcare interventions to encompass also approaches that address the socioeconomic determinants of mental health. Several authors have argued before that it is crucial to broaden the perspective on mental health and adopt population-level approaches that consider how the environments and wider societal structures in which people live affect mental health [33, 34].

However, achieving a socially just increase in NSES has shown to be difficult. In the Netherlands, there is a strong tradition of neighbourhood-based policies, and since the 1990s the focus has been on social mixing policies that change the composition of neighbourhoods [18]. Yet, previous studies demonstrate that the attempts of Dutch cities to adopt the mixed-neighbourhood approach to prevent segregation of low SES households in specific neighbourhoods have had limited effects [35]. One of the policies of this kind that has been highly contested is the Dutch Act on Extraordinary Measures for Urban Problems, also called The Rotterdam Act, legislation introduced in 2003 that allows municipalities to prohibit non-employed households from moving into social housing in targeted deprived areas. Some authors argue that this policy can be seen not only as exclusionary and unjust but also as ineffective in achieving the ultimate goal of increased NSES [36]. In the context of mental health, it is important to consider the impact of the mechanisms through which social mixing and increase in NSES are achieved. In this regard, exclusionary policies that displace or limit the lives of low-income residents would be unlikely to bring mental health benefits.

The National Program Rotterdam South is aimed at reducing the disparities between Rotterdam south and north neighbourhoods which is akin to Scenario 1 from our study, where the most socioeconomically deprived neighbourhoods (not necessarily only in the south, but anywhere in the city) were to reach the Rotterdam average NSES. The programme also aims to create socioeconomic conditions in Rotterdam South that are comparable to the conditions in the three other major Dutch cities, therefore bringing it closer to the national average. This programme focuses on three pillars: education, economic strengthening, and improvements in the physical quality of the neighbourhoods [21]. In contrast to traditional social mixing policies where the main aim is to change the composition of the neighbourhood population, this programme focused on improving the conditions for the individuals in the neighbourhood. Furthermore, prior studies show that the link between neighbourhood deprivation and depression strongly depends on individual-level SES, suggesting that individual-level SES is a key policy target factor in deprived neighbourhoods [8]. Therefore, a more equitable policy approach would involve implementing measures focused on lifting individuals out of poverty, preventing intergenerational transmission of poverty, and providing residents with access to meaningful employment and educational opportunities, particularly those residing in the most socioeconomically deprived communities.

Even though we did not explicitly model this scenario, another way to think about the potential policy approaches in the context of our findings is to consider policies that would reduce the strength of the association between NSES and mental health. This means that the intervention would target mechanisms that provide less favourable conditions for mental health in the lower SES neighbourhoods, such as poor-quality housing [37], reduced social cohesion, trust, safety, or collective efficacy [14].

There are several limitations to the HIA methodology that should be noted. First, we assume a causal relationship between the exposure and outcome, i.e. that NSES causes mental health problems. It has been demonstrated before that the relationship between SES and mental health may be bidirectional, meaning that mental health problems may cause financial problems that result in a lower SES [38]. However, despite the relationship being bidirectional, we should not dismiss the effect that increasing the SES would have on mental health, especially in the most deprived groups of society who are experiencing elevated distress [7]. Additionally, the ERF in our study is based on a pooled estimate from eight Dutch cohort studies where the outcome of interest was depression that was measured with various depression scales. The K-10 questionnaire that we used in the current analysis is a measurement for psychological distress or depression and anxiety and covers a broader spectrum of symptoms than only depression. However, since previous studies show that depression and anxiety have high comorbidity [39], we consider that it is reasonable to assume that the odds ratio estimate from Generaal *et al.* [6] is applicable to common mental health problems in general. For future research, we recommend replicating our study using another mental health scales as the outcome measures. For prior HIAs of physical health conditions, incident cases instead of prevalent cases are typically used because these are new cases each year that could be prevented if exposure was eliminated. In the present study, we only had prevalence data available for the HIA calculations; therefore, future studies with incidence data are highly recommended.

This novel application of the HIA methodology offers a highly policy-relevant estimation of how increasing NSES in the most socioeconomically deprived areas of the city could prevent mental health problems and reduce mental health inequalities between neighbourhoods. One of the main advantages lies in its capacity to apply scientific insights to a particular demographic, thereby enabling policymakers and other stakeholders to ascertain the scale of the affected population and the distribution across neighbourhoods. Although the study does not spell out how the increase in NSES can be achieved, it provides a rationale for improving socioeconomic conditions in the most vulnerable communities, namely, highlighting the mental health benefits that currently the most marginalized communities would gain. Furthermore, since health inequalities are observed for many health conditions, not just mental health, further research is needed on how increasing NSES could reduce cases of other diseases. As a whole, this would likely result in considerable benefits for public health.

## Supplementary Material

ckae222_Supplementary_Data

## Data Availability

Study data were sourced from several publicly available data repositories. The NSES score (2016) was requested via the GECCO database: https://www.gecco.nl/exposure-data-1/. The data on mental health per neighbourhood were downloaded from the publicly available Statline website, which is hosted by the Dutch National Institute for Public Health and the Environment (RIVM): https://statline.rivm.nl/#/RIVM/nl/dataset/50090NED/table. Rotterdam population data were sourced from the Municipality of Rotterdam databank: https://onderzoek010.nl/jive. Key points Prevalence of common mental health problems (depression and anxiety) is higher in neighbourhoods of lower socioeconomic status. Around 11% of annual cases of common mental health problems in Rotterdam could be prevented if the currently socioeconomically most deprived areas would reach the Rotterdam median NSES score. Around 20% of annual cases of common mental health problems in Rotterdam could be prevented if the currently socioeconomically most deprived areas would reach the Dutch national average NSES score. In both scenarios, inequalities in mental health between neighbourhoods would also be reduced. The findings of this novel application of the Health Impact Assessment methodology provide a rationale for further development of policies that improve socioeconomic conditions in cities.

## References

[1] World Health Organization. Mental disorders. https://www.who.int/news-room/fact-sheets/detail/mental-disorders (2 May 2024, date last accessed).

[2] GBD 2019 Mental Disorders Collaborators. Global, regional, and national burden of 12 mental disorders in 204 countries and territories, 1990–2019: a systematic analysis for the Global Burden of Disease Study 2019. Lancet Psychiatry 2022;9:137–50. 35026139 10.1016/S2215-0366(21)00395-3 PMC8776563

[3] United Nations, Department of Economic and Social Affairs, Population Division. World Urbanization Prospects The 2018 Revision. https://population.un.org/wup/Publications/Files/WUP2018-Report.pdf (2 May 2024, date last accessed).

[4] van der Wal JM , van Borkulo CD, Deserno MK et al Advancing urban mental health research: from complexity science to actionable targets for intervention. Lancet Psychiatry 2021;8:991–1000. 34627532 10.1016/S2215-0366(21)00047-X

[5] Marbin D , Gutwinski S, Schreiter S et al Perspectives in poverty and mental health. Front Public Health 2022;10:975482. 35991010 10.3389/fpubh.2022.975482 PMC9386343

[6] Generaal E , Timmermans EJ, Dekkers JEC et al Not urbanization level but socioeconomic, physical and social neighbourhood characteristics are associated with presence and severity of depressive and anxiety disorders. Psychol Med 2019;49:149–61. 29540253 10.1017/S0033291718000612 PMC6316373

[7] Richardson R , Westley T, Gariépy G et al Neighbourhood socioeconomic conditions and depression: a systematic review and meta-analysis. Soc Psychiatry Psychiatr Epidemiol 2015;50:1641–56. 26164028 10.1007/s00127-015-1092-4

[8] Neally SJ , Tamura K, Langerman SD et al Associations between neighbourhood socioeconomic deprivation and severity of depression: data from the National Health and Nutrition Examination Survey, 2011–2014. SSM Popul Health 2022;18:101111. 35601220 10.1016/j.ssmph.2022.101111 PMC9118884

[9] Aneshensel CS , Phelan JC, Bierman A. Handbook of the Sociology of Mental Health. Dordrecht: Springer, 2013.

[10] Putnam RD. Bowling Alone: The Collapse and Revival of American Community. New York: Simon & Schuster, 2000.

[11] Berger-Schmitt R. Considering social cohesion in quality of life assessments: concept and measurement. Soc Indic Res 2002;58:403–28. [Mismatch]

[12] Sampson RJ , Raudenbush SW, Earls F. Neighborhoods and violent crime: a multilevel study of collective efficacy. Science 1997;277:918–24. 9252316 10.1126/science.277.5328.918

[13] Corcoran R , Mansfield R, De Bezenac C et al Perceived neighbourhood affluence, mental health and wellbeing influence judgements of threat and trust on our streets: an urban walking study. PLoS One 2018;13:e0202412. 30114264 10.1371/journal.pone.0202412 PMC6095595

[14] Jakobsen AL , Jørgensen A, Tølbøll L et al Opening the black box of the relationship between neighborhood socioeconomic status and mental health: neighborhood social-interactive characteristics as contextual mechanisms. Health Place 2022;77:102905. 36096067 10.1016/j.healthplace.2022.102905

[15] Singh A , Daniel L, Baker E et al Housing disadvantage and poor mental health: a systematic review. Am J Prev Med 2019;57:262–72. 31326010 10.1016/j.amepre.2019.03.018

[16] Rao ND , Min J. Decent living standards: material prerequisites for human wellbeing. Soc Indic Res 2018;138:225–44. 29950752 10.1007/s11205-017-1650-0 PMC6013539

[17] Centraal Bureau voor de Statistiek. Statusscore per wijk en buurt o.b.v. welvaart, opleidingsniveau en arbeid, 2022. https://www.cbs.nl/nl-nl/achtergrond/2022/14/statusscore-per-wijk-en-buurt-o-b-v-welvaart-opleidingsniveau-en-arbeid (2 May 2024, date last accessed).

[18] Bolt G , van Kempen R. Neighbourhood based policies in the Netherlands: counteracting neighbourhood effects? In: Manley D, van Ham M, Bailey N, Simpson L, Maclennan D (eds.), Neighbourhood Effects or Neighbourhood Based Problems? A Policy Context. Dordrecht: Springer Netherlands, 2013, 195–213.

[19] Tran LD , Rice TH, Ong PM et al Impact of gentrification on adult mental health. Health Serv Res 2020;55:432–44. 31957022 10.1111/1475-6773.13264 PMC7240775

[20] Programmabureau Nationaal Programma Rotterdam Zuid. Nationaal Programma Rotterdam Zuid. Uitvoeringsplan 2023-2027. https://www.nprz.nl/ (2 May 2024, date last accessed).

[21] Deetman J , Mans W. Kwaliteitssprong Zuid. Ontwikkeling vanuit kracht. https://www.nprz.nl/over-nprz/onze-documenten/kwaliteitssprong-zuid-ontwikkeling-vanuit-kracht (2 May 2024, date last accessed).

[22] Mueller N , Rojas-Rueda D, Basagaña X et al Urban and transport planning related exposures and mortality: a health impact assessment for cities. Environ Health Perspect 2017;125:89–96. 27346385 10.1289/EHP220 PMC5226698

[23] Yañez DV , Pereira-Barboza EP, Cirach M et al An urban green space intervention with benefits for mental health: a health impact assessment of the Barcelona “Eixos Verds” Plan. Environ Int 2023;174:107880. 37002012 10.1016/j.envint.2023.107880

[24] Khomenko S , Cirach M, Pereira-Barboza E et al Premature mortality due to air pollution in European cities: a health impact assessment. Lancet Planet Health 2021;5:e121–34. 33482109 10.1016/S2542-5196(20)30272-2

[25] Gemeente Rotterdam. Onderzoek010—Bevolking per 1 januari—2016—Buurten. https://onderzoek010.nl/jive (2 May 2024, date last accessed).

[26] Rijksinstituut voor Volksgezondheid en Milieu. StatLine—Gezondheid per wijk en buurt; 2012/2016/2020 (indeling 2020). https://statline.rivm.nl/#/RIVM/nl/dataset/50090NED/table (2 May 2024, date last accessed).

[27] Rijksinstituut voor Volksgezondheid en Milieu. Health Monitor for Adults and the Elderly, 2016. https://www.monitorgezondheid.nl/gezondheidsmonitor-volwassenen-en-ouderen/2016 (2 May 2024, date last accessed).

[28] Kessler RC, , Andrews G, , Colpe LJ et al Short screening scales to monitor population prevalences and trends in non-specific psychological distress. Psychol Med 2002;32:959–76. 10.1017/s003329170200607412214795 12214795

[29] Chen T , Guestrin C. XGBoost: a scalable tree boosting system. In: *Proceedings of the 22nd ACM SIGKDD International Conference on Knowledge Discovery and Data Mining, San Francisco, CA, USA, 13–17 August 2016*. New York: Association for Computing Machinery, 2016, 785–94.

[30] GECCO. Available exposure data. Socio-economic status scores (SES). https://www.gecco.nl/exposure-data-1/ (2 May 2024, date last accessed).

[31] Zhang J , Yu KF. What’s the relative risk? A method of correcting the odds ratio in cohort studies of common outcomes. JAMA 1998;280:1690–1. 9832001 10.1001/jama.280.19.1690

[32] Wilkinson L. ggplot2: elegant graphics for data analysis by Wickham, H. Biometrics 2011;67:678–9.

[33] Cutrona CE , Wallace G, Wesner KA. Neighbourhood characteristics and depression: an examination of stress processes. Curr Dir Psychol Sci 2006;15:188–92. 18185846 10.1111/j.1467-8721.2006.00433.x PMC2186297

[34] Hogan AJ. Social and medical models of disability and mental health: evolution and renewal. CMAJ 2019;191:E16–E18. 31009368 10.1503/cmaj.181008 PMC6312522

[35] Koster HRA , van Ommeren J. Neighbourhood stigma and place-based policies. Econ Policy 2024;38:289–339.

[36] van Gent W , Hochstenbach C, Uitermark J. Exclusion as urban policy: the Dutch ‘act on extraordinary measures for urban problems’. Urban Stud 2018;55:2337–53. 30443086 10.1177/0042098017717214 PMC6195158

[37] Curl A , Kearns A, Mason P et al Physical and mental health outcomes following housing improvements: evidence from the GoWell study. J Epidemiol Community Health 2015;69:12–9. 25205160 10.1136/jech-2014-204064

[38] Boderie NW , van Kippersluis H, Been JV et al Examining neighbourhood effects on mental health utilizing a novel two-stage modeling approach. Ann Epidemiol 2023;83:60–70.e7. 37100099 10.1016/j.annepidem.2023.04.012

[39] ter Meulen WG , Draisma S, van Hemert AM et al Depressive and anxiety disorders in concert—a synthesis of findings on comorbidity in the NESDA study. J Affect Disord 2021;284:85–97. 33588240 10.1016/j.jad.2021.02.004

