# Supplementary material for: Preventing common mental health problems by increasing neighbourhood socioeconomic status: a mental health impact assessment in Rotterdam, the Netherlands
Source: Eur J Public Health. 2025 Jan 13;35(1):72–8. doi: 10.1093/eurpub/ckae222 (PMC11832156; doi:10.1093/eurpub/ckae222)
Supplement: ckae222_Supplementary_Data [file ckae222_supplementary_data.docx]

Supplementary material

**Article:** Preventing Common Mental Health Problems by Increasing Neighbourhood Socioeconomic Status: A Mental Health Impact Assessment in Rotterdam, the Netherlands

**Authors:**

Daina Kosīte^1^ <https://orcid.org/0009-0005-6608-4547>

Frank J van Lenthe^1^ <https://orcid.org/0000-0001-6402-7075>

Mark J Nieuwenhuijsen^2^ <https://orcid.org/0000-0001-9461-7981>

Mariëlle A Beenackers^1^ <https://orcid.org/0000-0002-7981-3391>

Step 1: Calculate the exposure difference (ED) between the baseline and counterfactual NSES scores

$$ED=\left( {NSES}_{counterfactual} \right)-\left( {NSES}_{baseline} \right)$$

Step 2: Apply the exposure response function (ERF) to scale the relative risk (RR) within each neighborhood

$${RR}_{ED}=e^{\left[ \frac{log(RR)}{1} \times ED \right]}$$

Step 3: Calculate the Population Attributable Fraction (PAF) in each neighborhood

$$PAF=\frac{({RR}_{ED}-1)}{{RR}_{ED}}$$

Step 4: Calculate the preventable cases (PC) per neighborhood based on the baseline prevalence of common mental health problems (P)

$$PC=PAF \times P_{mental\_health}$$

**Figure S1** HIA Procedure based on the UTOPHIA procedure.


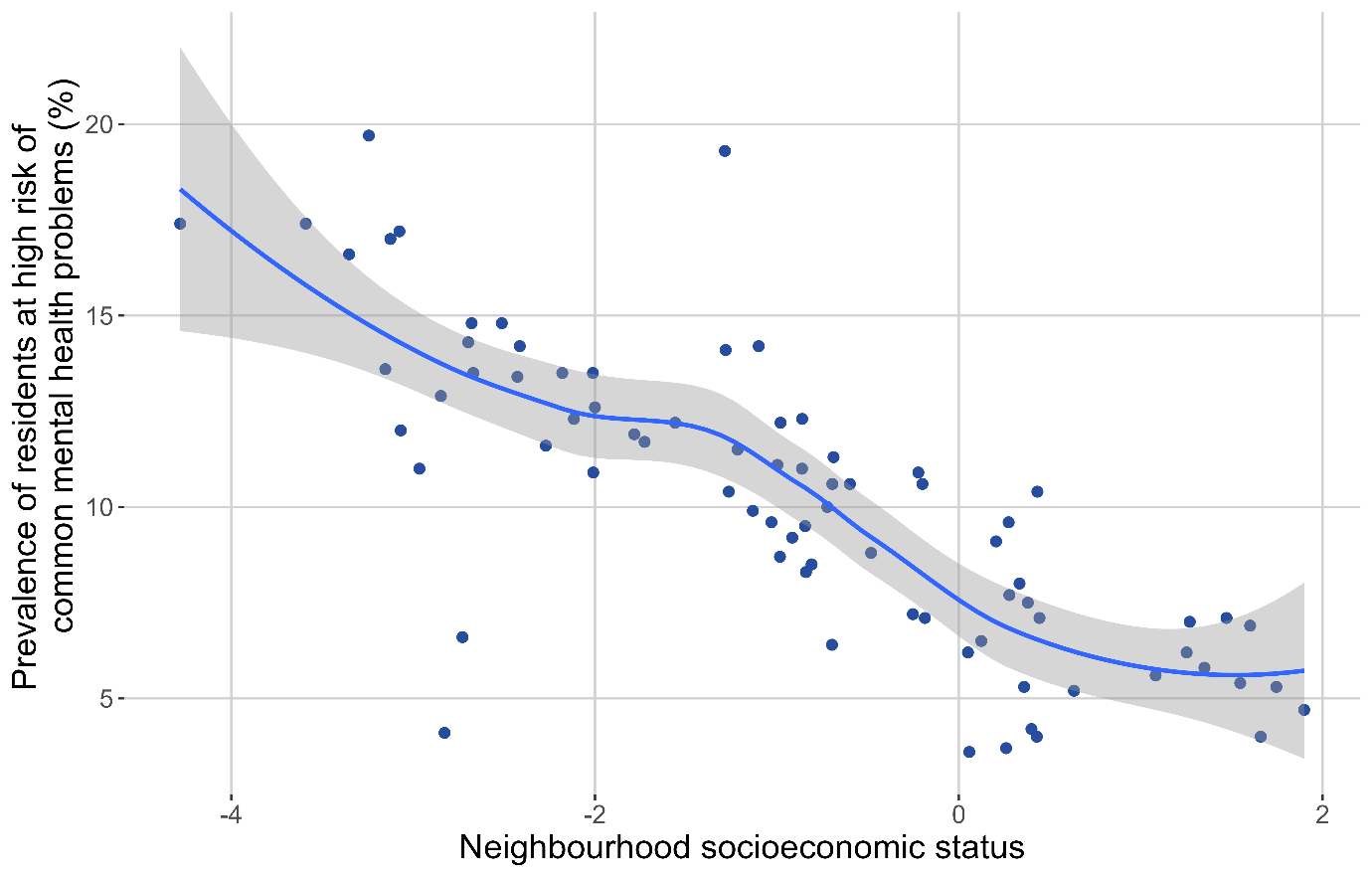
**Figure S2** Relationship between neighborhood socioeconomic status and prevalence of mental health problems in Rotterdam
